# Addressing health disparities and equitable representation in cancer research through the initiative, ‘The Patient and Public Involvement Cancer Research Group for Diverse Backgrounds’

**DOI:** 10.3332/ecancer.2025.1885

**Published:** 2025-04-10

**Authors:** Saran Green, Aida Santaolalla, Beth Russell, Gincy George, Harriet Wylie, Maria Monroy-Iglesias, Ailbhe Lawlor, Mark Minott, Annette Carty, Vernon Bailey, Tene Green, Zhane Peterson, Charlene Young, Mieke Van Hemelrijck

**Affiliations:** 1 Comprehensive Cancer Centre, King’s College London, London SE1 9RT, UK; 2 Patient and Public Involvement Cancer Research Group for Diverse Backgrounds, Comprehensive Cancer Centre, 3rd Floor Bermondsey Wing, Guy’s Hospital, London SE1 9RT, UK

**Keywords:** cancer research, patient and public involvement, patient engagement, equality diversity and inclusion, underrepresentation, resource tool

## Abstract

This paper addresses the persistent underrepresentation of Black, Asian and minority ethnic communities in health research, particularly in the context of prostate cancer. Despite the disproportionately higher burden of cancer in Black, Asian and minority ethnic communities, their representation in research remains inadequate. This paper highlights the efforts of The Patient and Public Involvement Cancer Research Group for Diverse Backgrounds (Diverse PPI), a PPI group established to address this disparity. We describe the rationale behind Diverse PPI’s formation, emphasising the need for targeted outreach and culturally competent practices. The group’s activities include organising public engagement events, developing resources such as ARUARES (a tool designed to foster inclusive research) and collaborating with researchers on various projects. The paper illustrates how Diverse PPI has made a positive impact through collaborative working, empowering diverse ethnic communities and advocating for inclusive research practices. We conclude by emphasising the importance of similar initiatives in addressing health disparities and achieving equitable representation in cancer research.

## Background

**Figure 1.**
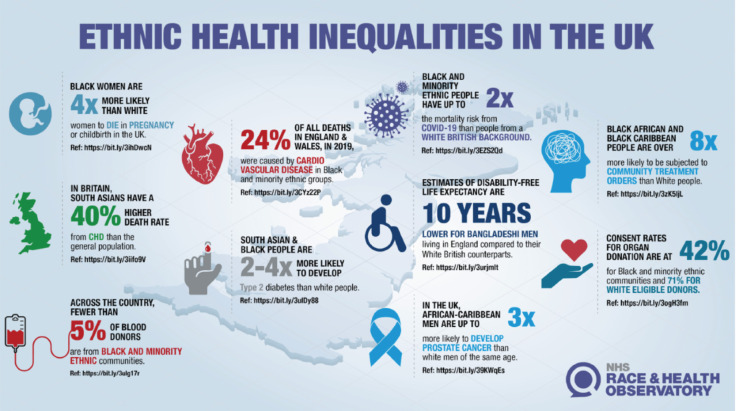
Ethnic health inequalities in the UK.

Despite the higher incidence and worse mortality rates among Black, Asian and minority ethnic communities in several diseases, their representation in research remains disproportionately low (Figure 1). The National Health Service Race and Health Observatory highlights ethnic health disparities in the UK, exemplified by African–Caribbean men having up to three times higher prostate cancer (PC) rates than their White counterparts [1].

A multitude of interconnected reasons, ranging from mistrust of research based on previous health injustices to logistical challenges such as transportation and work, contribute to the underrepresentation of specific groups in health research [2]. Unravelling the reasons for the low representation of diverse ethnic groups reveals the interplay of social, cultural and historical dynamics that influence research engagement in these communities [3]. Without comprehensive data from diverse populations, addressing health disparities will be an ongoing challenge [3], a reality magnified and amplified by the COVID-19 pandemic [4].

Despite commendable efforts, progress towards diverse clinical trials remains slow [5]. This lack of inclusivity has significant consequences, as their effectiveness remains unclear if tested only on a restricted population. Genetic variations can dramatically impact treatment responses and limiting trial participants can lead to treatments that do not work equally well for everyone [6].

Recognising this crucial issue, the United States Food and Drug Administration took a fundamental step in April 2022 towards ensuring fairer and more effective treatments for all. This new law mandates sponsors to actively plan for the inclusion of different racial, ethnic, gender and age groups in their studies, promoting fairness and equal access to potential new treatments. This legal mandate is a significant step towards achieving clinical trials that reflect the wider population, ultimately leading to more accurate data and, consequently, better treatments for everyone [6].

The importance of diversity in clinical research is further exemplified by the case of PC. Historically, the majority of PC cell lines used in research are from White men, resulting in a limited understanding of how the disease affects other populations [7]. This narrow focus missed an important gene variation impacting Black men’s response to a common PC drug. This is particularly concerning considering the higher prevalence of PC in Black men. Diversifying cell lines and study participants is essential to uncover such biological differences and develop truly effective therapies for all [7].

The ReIMAGINE Consortium aims to, for the first time, combine biomarkers (blood, urine and tissue) with state-of-the-art imaging to predict which PCs are likely to rapidly progress over time and which will not. To ensure that PC patients and their families remain the focus of the study, ReIMAGINE involved both parties at all stages of the research process. By integrating patient and public voices in every research stage, we were able to design studies that resonated with participants, easy to join and ultimately delivered more meaningful results [8].

Since the initiation of the project, the ReIMAGINE Consortium has been working with patients and the public to ensure that PC research is relevant to men and their families. We had several meetings to try to ensure that the ethnic diversity of London was represented in the study. These meetings were scheduled alternatively during the daytime and in the evening, to allow a wide range of patients and members of the public to engage.

However, despite our efforts to represent London’s ethnic diversity and make the study as diverse as possible, the volunteers from Black, Asian and minority ethnic communities represented in the meetings ranged from 0% to 13% – even though the risk of PC is higher among Black men. Therefore, our team took proactive measures recognising the need for targeted outreach, particularly for Black men.

A patient and public involvement (PPI) Coordinator was appointed to work closely with the patient representative to recruit a diverse PPI Sub-Committee. The active work of the ReIMAGINE PPI Sub-Committee hence led to work outside the study remit, particularly in the establishment of a Black, Asian and Minority Ethnic PPI cancer research group.

The current paper is a small snapshot of how working in partnership with diverse communities enabled meaningful outputs. Our goal is to raise awareness and advocate for better representation in research, not to blame or discriminate against any specific group, providing solutions and highlighting the shared goal of better healthcare for all. We describe our collaborative efforts – bringing together all stakeholders through productive and inclusive dialogue – taking action towards bringing about positive change through various initiatives.

## The initiative: PPI cancer research group for diverse backgrounds (Diverse PPI)

Established in 2020, Diverse PPI evolved from a PC-focused group to a forum for diverse ethnic communities affected by any cancer with specific aims and objectives:

1. To create a research forum that represents the voices of those from diverse communities directly affected by research projects and healthcare services.
2. To provide a platform for researchers to access this under-represented population of patients and members of the public who wish to be involved with clinical research.
3. To provide a greater range of perspectives from the Black, Asian and minority ethnic communities in research.
4. To empower, educate and equip diverse ethnic communities, so they can make informed decisions about their health and engage with research.

## Collaborative working

Diverse PPI has been working to address the disproportionate under-representation of the Black, Asian and minority ethnic communities in research since its establishment, contributing to numerous studies nationally and globally, garnering appreciation from international researchers and local institutions. For instance, Diverse PPI provided a focus group for researchers from Milan who were writing a proposal for the *World Cancer Research Fund,* requesting ethnic representation in a focus group. In addition, colleagues from the Royal Marsden Hospital were grateful for the group providing Black representation in their focus group with PC patients: ‘He was great, fabulous contribution, so worthwhile.’

Mark Minott, an active member of our specialist group states, *‘I joined the group because of my experience with PC. More importantly, joining the group provides an opportunity to use my research skills coupled with my cultural background to help influence research that benefits the BAME community.*’

## Group activities

### Public engagement event

Diverse PPI continues to empower communities and foster engagement in research activities. In April 2023, they organised ‘About us, By us…,’ a face-to-face public engagement series at Guy’s Hospital. This initiative aimed to create an inclusive space, enabling discussions on barriers, experiences and solutions related to engaging with diverse communities.

It was important for the members to produce an event about them and by them – understanding their communities and how to tailor involvement and engagement. The aim was to create a forum to foster sharing and learning needed to guide and support all participants; patients, members of the public, healthcare professionals as well as academics.

Healthcare professionals and academics had the opportunity to hear from the patients and members of the public, about the barriers engaging with diverse communities, identifying practical actions for change and providing practical solutions to engagement with diverse communities. This involved discussions on barriers, such as culture and mistrust. The members shared their experiences on topics such as ‘privacy’ in the Black community and ‘honour’ in the Asian community.

Similarly, the team encouraged patients and the public from diverse communities to engage with health research by highlighting the gaps in data and the benefits of taking part, such as, contributing to improvements in patient care, curriculum vitae/resume development, confidence building, supplementary income and so on. They discussed the local and international public authorities, health agencies, governing bodies, (ethical) frameworks set up to safeguard them and their data. Furthermore, they addressed the warranted issues of mistrust and how PPI is attempting to level the playing field.

The special guest speaker was Professor Shaun Treweek from the University of Aberdeen. Prof Treweek is a health services researcher interested in efficient trial design, particularly around inclusive recruitment and retention and the effective presentation of research evidence. He reinforced the group’s points and shared some of the frameworks which he has led the development of, for example, the NIHR INCLUDE Ethnicity Framework – a tool to help trialists design inclusive trials, and PRECIS-2 – a tool to match trial design decisions to what the users of the results need [9].

It was an evening of informative talks and lively discussions in a safe space, where diverse perspectives were represented. Finding mutually beneficial solutions and ensuring it was a positive learning experience for all attendees were key to the members. Given the success of ‘About us, By us…’ the group has plans to continue the event as a series in the future.

### Resource tool

**Figure 2.**
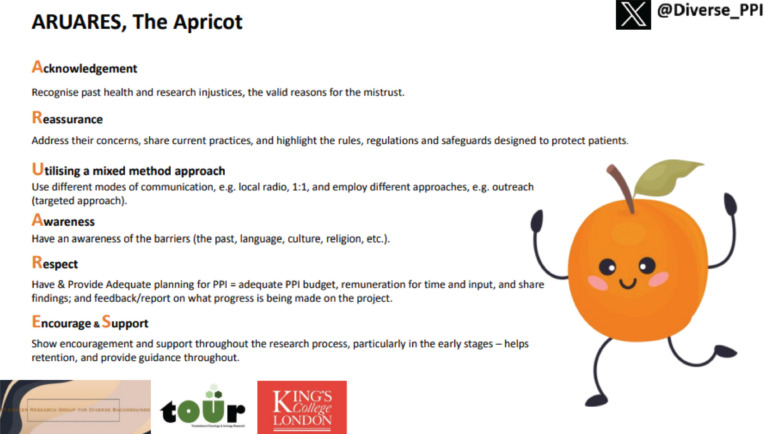
ARUARES, the Apricot - a resource tool to support researchers in engaging diverse communities.

Diverse PPI developed a simple, yet powerful and innovative resource, ARUARES, The Apricot, to support researchers engaging diverse communities with no additional costs or resources. The resource was introduced at their first public engagement event in April 2023, ‘About us, By us…’ ARUARES, The Apricot is an acronym that serves as a mental reminder when seeking to engage diverse communities. The members have shown that inclusivity does not always require extra resources, but rather mindful consideration. ARUARES, The Apricot, is free and can be downloaded from the King’s College London Transforming cancer Outcomes through Research (TOUR) team’s webpage (Figure 2). Currently, the resource has been utilised by various research groups nationwide including the Institute of Cancer Research, The Royal Marsden, Queen Mary’s and the University of Surrey.

### Diverse PPI initiative outcomes to date

Through its various PPI activities and engagement events, including community outreach, Diverse PPI has been equipping diverse communities with knowledge and the skills to engage with health and health research through building trust and raising awareness. They have hosted information and awareness stands and stalls at various local events, sharing the safeguards that are in place today to protect patients and their data and raising awareness about the lack of comprehensive data from diverse populations.

**Figure 3.**
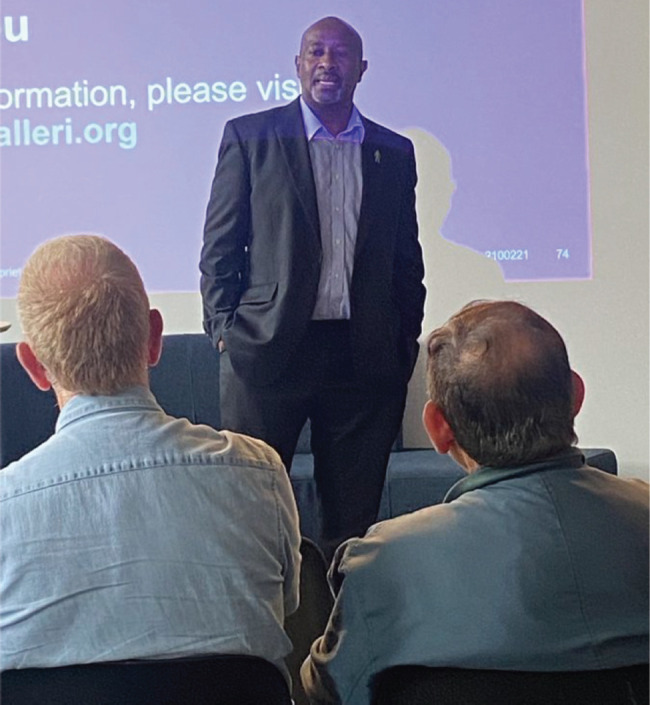
Vernon Bailey, active member of the PPI cancer research group for diverse backgrouds.

**Figure 4.**
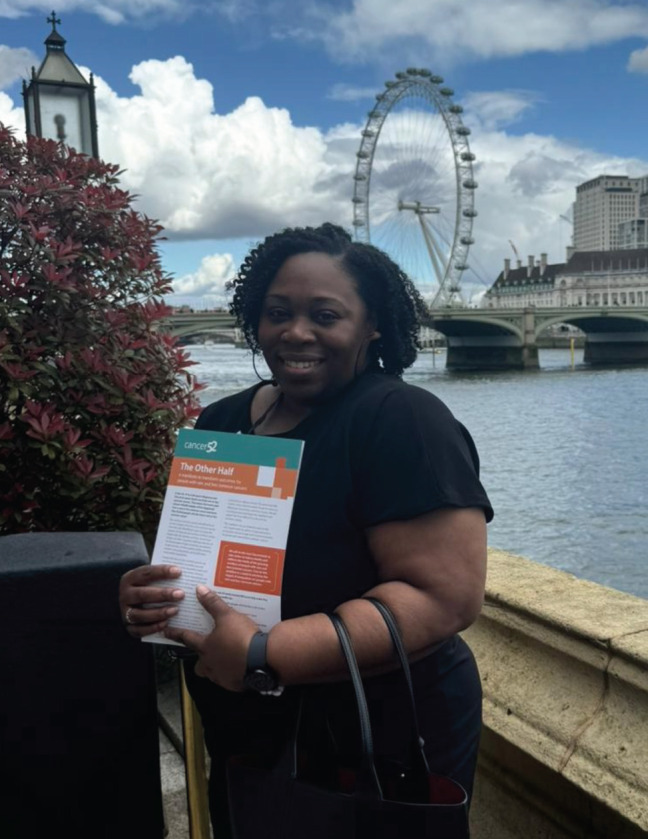
Charlene young, active member of the PPI cancer research group for diverse backgrounds.

Many of its members are now sharing their knowledge and skills with other groups and networks independent of Diverse PPI. Vernon Bailey (Figure 3), an active member of the group, has been advocating for representation in research at different events and has appeared in different communication initiatives such as a video for the NHS’ National Patient Experience Survey and the South East London Consumer Alliance’s video promoting conversations about PC in the black community. Furthermore, in April 2024 Charlene Young (Figure 4), an established patient advocate, attended parliament for the launch of C52Manifesto to raise awareness of rare cancers to improve treatments and outcomes. Diverse communities seeing representation from people who look like them have motivated others from the community to be involved in research. The group has developed a reputation within the research/healthcare community and are often approached to support various NHS, research initiatives for example ‘Navigating digital health: a guide to data and artificial intelligence in healthcare’. Many studies have revised their study design and protocols to include culturally and linguistically appropriate language as per the advice of the group.

*‘The Diverse PPI group has been instrumental in advising and guiding my research. For instance, the group provided invaluable feedback on the recruitment process, and this resulted in an enhanced strategy and a tailored and culturally appropriate recruitment poster that maximised the opportunities to reach and engage participants in the study’*

[Mar Estupiñán Fdez. de Mesa, Postgraduate Researcher PhDc, University of Surrey]

*‘Diverse PPI’s contribution has been very relevant and useful. The focus and coverage of my research was changed from focusing mainly on Black African women to incorporate the Caribbean women. A notable change which was also recorded in the research protocol.’*

[Anietie Aliu, PhD Student, University of Surrey]

Diverse PPI not only promotes the benefits of engaging with research in their respective communities, they also highlight the impact of previous health and research injustices on engagement with diverse groups of healthcare professionals and researchers. In addition to providing practical considerations when engaging these populations such as logistical challenges, for example, transportation, childcare costs and so on, at different public engagement events, workshops and panel discussions. In February 2024, Diverse PPI were invited to be panelists at Cancer Research UK’s Annual Symposium at Imperial College London discussing enhancing EDI in participatory research.

## Lessons learned

Diverse communities are riddled with apprehensions and reticence toward health research. Developing meaningful relationships in underrepresented communities/groups requires adequate time and commitment to build community trust via the community. This involves feet on the ground and allocating adequate time and sufficient resources for meaningful community outreach. Diverse PPI which is sponsored by the TOUR team has invested time and money traveling as well as participating in different community events to network and build lasting relationships.

Like the name of Diverse PPI’s engagement event ‘About us, By us’ suggests, nothing about them without them, this is the premise that all should take when seeking to engage diverse groups. Working in partnership is crucial to the success of any PPIE initiative. Giving public contributors ownership of the initiative is empowering and fosters commitment. No one party should have sole power over the decision-making process. All stakeholders should have equal opportunity to contribute and to be involved. ‘About us, by us’ was a successful initiative with high impact due to codesign and coproduction with all stakeholders.

Successful coproduction requires flexibility from both parties. Therefore, efforts must be made to remove existing barriers to participation and using a range of inclusive methods and technologies to support engagement. For ‘About us, By us,’ the working group was established, consisting of some members and staff from the TOUR team to coordinate and support the ongoing process. The working group meetings were held in person and online to accommodate all participants as well as resources were provided both in print and via email.

## Conclusion

Understanding the reasons behind low research participation from diverse ethnic communities requires acknowledging the historical context, cultural perceptions and practical challenges faced by different communities. Increased involvement from diverse communities in cancer research is crucial for acquiring valuable data and genetic information, to ultimately improve health outcomes for all. Hence, more groups like Diverse PPI and similar initiatives are needed to educate, equip and empower Black, Asian and minority ethnic communities, to engage with their health and health research and address health disparities. Actively working towards building trust and adopting culturally competent practices to recruit and engage ethnic minorities in research is imperative for closing the health gap.

For more information on Diverse PPI, please direct message the group on ‘X’ formally known as Twitter: @Diverse_PPI; or on Bluesky: @diverseppi.bsky.social.

## Conflicts of interest

The authors declare that they have no conflicts of interest.
